# Floor of the Mouth Hemorrhage Following Dental Implant Placement or Guided Bone Regeneration (GBR) in the Atrophic Interforaminal Mandible

**DOI:** 10.1155/crid/8413875

**Published:** 2024-12-24

**Authors:** Domenico Sfondrini, Stefano Marelli, Rachele Patriarca, Andrea Scribante, Lorenzo Preda, Gabriele Savioli, Giorgio Novelli, Alessandro Bardazzi

**Affiliations:** ^1^Department of Maxillofacial Surgery, Fondazione IRCCS Policlinico S. Matteo, Pavia, Italy; ^2^Section of Dentistry, Department of Clinical, Surgical, Diagnostic and Pediatric Sciences, University of Pavia, Pavia, Italy; ^3^Institute of Radiology, Department of Diagnostic and Imaging Services, Fondazione IRCCS Policlinico San Matteo, Pavia, Italy; ^4^Emergency Department, Fondazione IRCCS Policlinico San Matteo, Pavia, Italy; ^5^Maxillofacial Surgery, Department of Medicine and Surgery, School of Medicine, IRCCS San Gerardo dei Tintori Foundation, University of Milano-Bicocca, Monza, Italy; ^6^Department of Maxillofacial Surgery, Azienda Ospedaliera Papa Giovanni XXIII, Bergamo, Bergamo, Italy

**Keywords:** bleeding, GBR major complication, implant surgery, mouth floor hemorrhage, oral surgery

## Abstract

The authors present two cases of mouth floor hemorrhage consequences of implant placement within the atrophic anterior mandible. In one patient, the implant placement was associated with the guided bone regeneration (GBR) technique. This serious complication has been widely described in the literature, especially in the anterior mandible area. In cases of bone resorption, the edentulous ridge becomes closer to the artery, and the risk of vessel injury increases. In both patients, the hematoma rapidly spread in the loose tissues of the mouth floor, displacing the tongue posteriorly and cranially, with airway compromise. The patients were hospitalized with nasotracheal intubation to secure the airway. In both patients, the bleeding stopped spontaneously, and after a few days, the oral floor swallowing was reduced, allowing the endotracheal tube to be removed. In about 2 weeks, the hematoma completely resorbed without surgery. According to the literature, the main cause of floor of the mouth hemorrhage is the mandibular lingual cortical plate perforation during bone drilling with subsequent sublingual–submental artery injury. In fact, in the first patient presented, this surgical error was clearly noticeable on the CT scan. Differently, in the second case reported, no radiological signs of inner cortical perforation were observed, and together with a mouth floor hematoma, a blood collection was also evident on the lower lip, suggesting a different cause of bleeding. Most likely during the periosteal release incision, mandatory in GBR technique, the ascending mental artery was injured, and hematoma spread in the mouth floor through the similar incision done on the lingual flap. Firstly, the mouth floor hemorrhage caused by an injury of a vestibular soft tissue artery during GBR surgery was reported. Strategies and recommendations to avoid this life-threatening event are provided, based on the literature review and the authors' experience.

## 1. Introduction

Endosseous dental implant placement, or even more invasive preimplantological procedures such as guided bone regeneration (GBR), is nowadays considered a safe and reliable technique. Nevertheless, they could lead to significant and severe complications not only during surgery but also in the postoperative period [1–3].

Floor of the mouth bleeding, as first reported by Krenkel and Holzner, is the most potentially life-threatening event [4, 5]. The hematoma can rapidly spread in the loose tissues of the mouth floor and displace the tongue posteriorly and cranially, with airway compromise. Hemorrhage control may require hospitalization and intubation or a tracheostomy to maintain the airway [6]. The incidence of this event is difficult to ascertain, and only a relatively few cases have been reported. No randomized controlled trials have been reported in the literature.

According to a systematic review by Balaguer-Martí et al. [5], the most frequent cause is perforation of the lingual cortical plate during bone drilling for implant placement, causing arterial blood vessel injury (mainly the sublingual artery). One case of periosteum tear during lingual flap elevation without any cortical bone interruption was also reported as the assumed reason for bleeding in the mouth floor hematoma [7].

The authors present two cases of hemorrhage consequences of dental implant placement within the atrophic anterior mandible. Both patients were treated by two different unraveled dentists and, a few hours after discharge from the outpatient clinic, spontaneously called for emergency services. To date, information about implant treatment (from planning to execution) and dental practitioners' skills remain unknown.

In the first patient, improper implant placement in a knife-edge ridge (a clear example of clinical malpractice) led to perforation of the lingual cortical plate with vessel injury.

In the second case, the implant placement was correctly associated with the GBR technique because of the insufficient mandibular bone thickness. No cortical perforations were seen on the CT scan, and the fixtures were well covered with alloplastic materials and resorbable membrane. The presence of a massive hematoma in the buccal side of the mandibular symphysis suggested an injury to the ascending mental artery as a reason for the bleeding. To the best of the authors' knowledge, no reports of mouth floor hematoma for vestibular soft tissue damage during GBR have yet been reported.

## 2. Cases Presentation

### 2.1. Case No. 1

**Figure 1:**
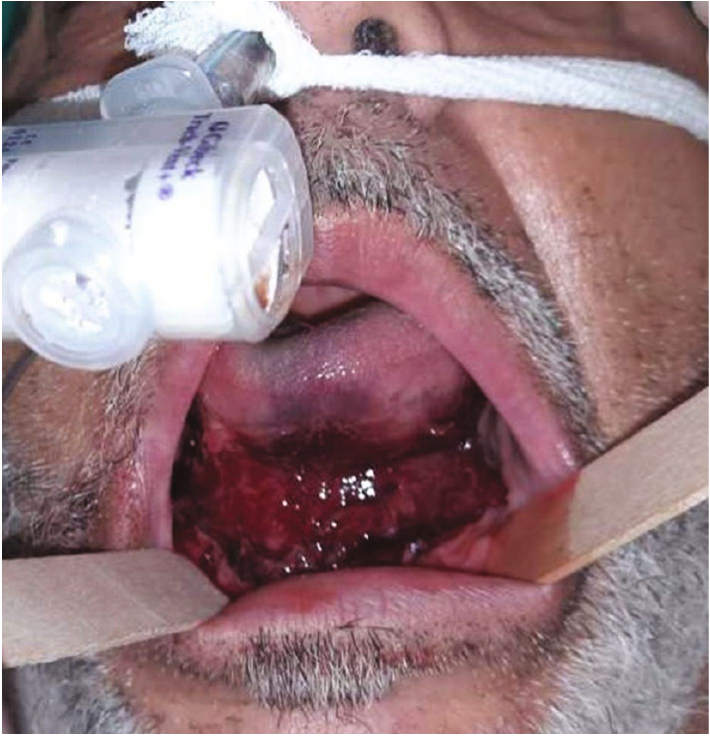
Mouth floor hematoma and nasotracheal tube in place.

**Figure 2:**
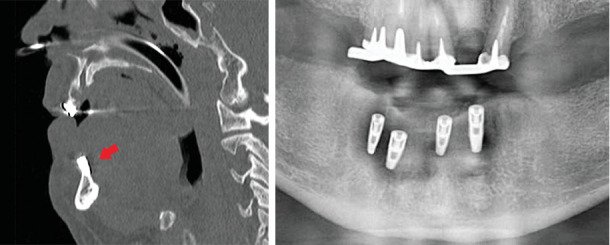
CT scan sagittal view. Nasotracheal tube with mouth floor and tongue displacement. The red arrow shows dehiscence at the lingual aspect of the fixtures. The OPT shows four implants incorrectly placed in the atrophic alveolar ridge.

A 52-year-old male patient underwent placement of four dental implants in the interforaminal region in a private dental office. Written approval and informed consent were obtained. Once discharged, he started to complain of swelling of the tongue with elevation of the floor of the mouth, leading to airway obstruction. The mobile emergency unit performed prompt tracheal intubation and prevented death by asphyxia. The patient was transferred to the ICU of IRCCS Policlinico San Matteo, and angiography with a CT scan was performed. No active bleeding was reported; the robust extravascular clots, reducing perivascular space, brought spontaneous hemostasis. Three days later, the nasotracheal tube was removed, and the patient was transferred to the maxillofacial unit for additional monitoring. Clinically, the hematoma was mainly located at the mouth floor (Figure 1), and the orthopantomography (Figure 2) revealed four implants placed in suspected horizontally compromised alveolar ridges (knife edge ridges).

The CT scan confirmed the insufficient mandibular thickness with lingual cortical plate absence around two implants (Figure 2), suggesting the blind drilling could have caused a vascular injury at the mouth floor.

The patient was discharged in good condition and, at 15 days follow-up, showed complete reabsorption of the hematoma.

The patient was treated by an external dental office, and unfortunately, no information regarding the surgical intervention, implant protocol followed, initial anatomical conditions, etc., is available yet. By measurement on a CT scan, the implant length ranges from 13 to 10 mm with a 3.5 mm diameter.

Based on the postsurgical CT scan, it can be stated that the implants were incorrectly placed in an insufficient ridge thickness without any previous or concomitant bone augmentation procedure. The presence of several implant dehiscences and fenestrations (Figure 2), which compromise the success and survival rate [8], is a clear indication of malpractice with ethical and legal implications.

### 2.2. Case No. 2

**Figure 3:**
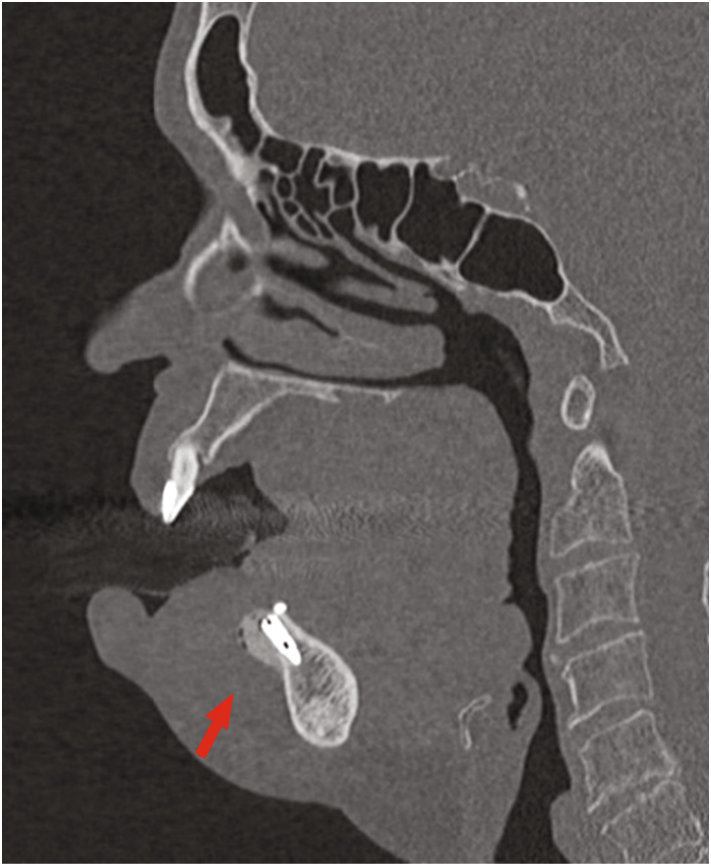
CT sagittal section after removing nasotracheal tube: fixture with the correct axis of insertion without lingual cortical perforation. The buccal hematoma is still present, whereas the tongue is less displaced. The red arrow shows horizontal GBR with bone grafting materials.

A 69-year-old woman came to our attention showing complications resulting from a GBR procedure simultaneous to dental implant placement in the symphyseal mandibular region. Written approval and informed consent were obtained. The dental surgery was performed in a dentistry outpatient clinic under local anesthesia, without intraoperative complications noticed. The patient was discharged home and a few hours later reported copious bleeding and swelling of the floor of the mouth that displaced the tongue cranially, leading to progressive airway obstruction. She was rushed to a local emergency department, where angiography and a CT scan were performed, showing active bleeding in the anterior mandibular region. A nasotracheal intubation was promptly achieved to protect the airways, then the patient was transferred to the ICU of IRCCS Policlinico San Matteo. Due to local clinical stability, a magnetic resonance (MR) angiography and a CT scan were performed about 12 h later, confirming no more active bleeding and an unchanged dimension of hematoma. Therefore, no surgical intervention or additional procedures were needed. The CT scan showed a previous GBR treatment with bone substitute material along with two dental implant placements in the anterior mandibular ridge. No. 2 bone tack miniscrews were visible at the lingual aspect of the alveolar ridge (Figure 3). At the buccal side, a wide hematoma was present, with mouth floor involvement and cranial displacement of the tongue. No lingual cortical perforation was observed, and the implants were well-oriented with the right axis of insertion (Figure 3).

**Figure 4:**
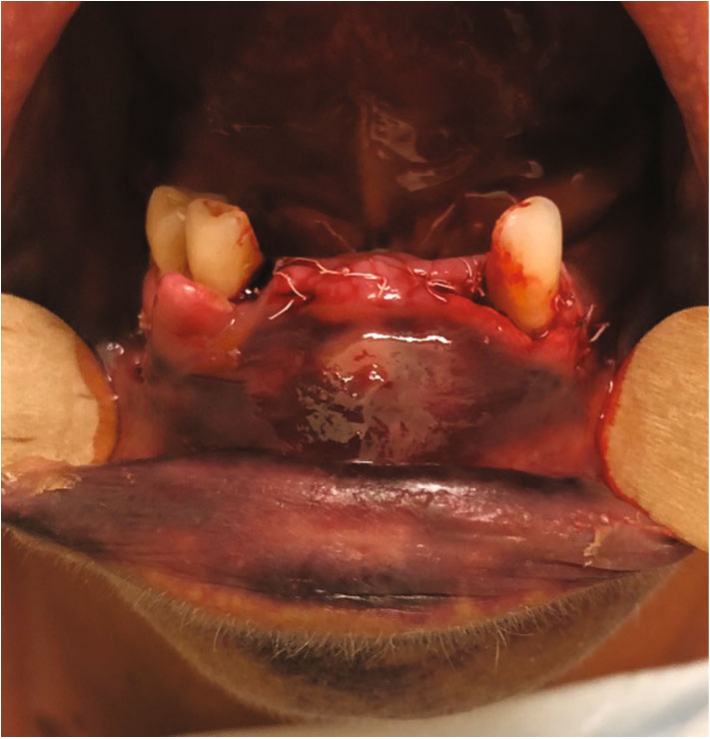
Five days after implant surgery. The lingual hematoma was considerably reduced. At the buccal side, a large hematoma was still present under the mucosa.

Three days later, the nasotracheal tube was removed, and the patient was transferred to our maxillofacial unit for additional monitoring. Clinically, the surgical wound was found almost completely closed despite the large hematoma at the vestibular aspect of the mandibular symphysis. The lingual hematoma and the mouth floor edema were still present even if markedly reduced (Figure 4).

After 72 h, she was dismissed in good clinical condition. One week later, during the follow-up, the patient showed pus discharge through the vestibular surgical wound; as suspected, the residual hematoma along with xenograft and resorbable membrane became infected. Antibiotic therapy was prescribed for 7 days with some symptom improvements.

After 2 weeks, the patient was referred to his own dentist for a surgical toilet. One month later, the wound was completely healed and closed. Afterwards, she was lost to follow-up.

The presence of wide hematoma mostly in the buccal aspect of the mandibular symphysis along with the absence of radiological signs of lingual cortical perforation (Figure 3) suggests the cause of bleeding was an injury of the ascending mental artery. According to the authors, the deep periosteal releasing incision, performed to advance the flap sufficiently for a tension-free flap closure in bone augmentation procedures, is assumed to have been the cause of vessel injury. The hematoma involved later even the mouth floor, passing over the mandibular ridge and through the periosteal incision of the lingual flap. This is the first case of mouth floor hemorrhage described as a major complication of the GBR technique.

## 3. Discussion

The anterior region of the mandible is a highly vascularized area. It is supplied by a rich anastomosing plexus among the sublingual branch of the lingual artery, the submental branch of the facial artery, and the incisive branches from the inferior alveolar artery [9].

This vascular plexus lies very close to the interforaminal lingual cortical plate of the mandible; therefore, accidental penetration of the anterior mandibular inner cortex and/or laceration of the adjacent soft tissues during minor oral and implant surgery may damage it, causing hemorrhages and potentially life-threatening situations [1, 10, 11]. In fact, the loose adipose and connective tissue in the floor of the mouth region does not constitute an effective barrier capable of containing the blood flow, even in case of small artery rupture. Therefore, an eventual hematoma can spread to the neighboring submandibular space and the tongue, causing upper airway obstruction.

During implant surgery in the mandibular interforaminal region, the vessel most often injured, causing the floor of mouth hemorrhage, is the sublingual artery with its distal branches [5].

This 2-mm-diameter artery arises from the lingual artery and courses between the genioglossus and mylohyoid muscles, running toward the lingual anterior cortical plate of the mandible and perforating it through the middle lingual canal (MLC) and the lateral lingual canal (LLC). Several branches of the sublingual artery connect with a deeper vessel, the submental artery.

This branch of the facial artery runs anteriorly on the surface of the mylohyoid muscle inferior to the body of the mandible and deep to the digastric muscle [9, 12–14].

Because of its deeper course, it is rarely injured during interforaminal implant surgery.

Once this vessel reaches the border of the mandibular symphysis, it turns superiorly toward the lip.

This terminal branch, called the ascending mental artery, curves to the chin and joins the arterial circulation of the lip. This artery was most likely the one injured in the first patient presented.

As reported in the literature, hemorrhage may start immediately or with some delay after vascular injury [15, 16]. Reflective constriction and retraction of a transected artery, along with the vasoconstricting effect of the epinephrine contained in the local anesthetic solution, may enable the formation of a blood clot and bleeding cessation. The delayed postoperative onset of hemorrhage may be linked to the compensatory vasodilation of the injured vessel due to a gradual absorption of epinephrine, loss of the initial thrombus, and resumptive bleeding [1].

In case of an early occurrence of massive submucosal hemorrhage, basic measures should be taken. Most important is immediate bimanual compression at the suspected perforation site and transport of the patient to the nearest hospital to secure the airway without delay [9, 16]. The airway control options include close observation, oro or nasotracheal intubation, and tracheostomy or cricothyroidotomy.

Once the airway is secured, efforts are undertaken for definitive resolution of hemorrhage. In most cases, as happened in both patients presented, hemostasis results from compression by the expanding hematoma, when the pressure of the extravasated blood equals the vascular pressure of the injured vessel [1].

Because the two patients showed stable hematoma without active bleeding, the authors preferred not to treat it; surgical drainage may promote further bleeding by lowering the established pressure.

Generally, surgical treatment is preferred when the hematoma is large and nonrestrictive [1]; surgical evacuation and ligation of the bleeding artery both with an intra- or extraoral approach permit a rapid resolution. Endovascular angiography is an alternative diagnostic tool permitting overcoming unsuccessful attempts to define and isolate the bleeding source, which can be then occluded by embolization [1, 17, 18].

Interestingly, both patients presented share the same anatomical characteristic: a horizontally compromised alveolar ridge with insufficient width (knife edge ridge). The interforaminal bone resorption pattern at the buccal side of incisor-canine areas can make conventional implant placement more difficult or impossible. In these cases, additional bone augmentation techniques for bone reconstruction, like bone grafting or GBR, are needed.

Vertical bone resorption reduces the distance between crestal bone and MML-LLC level, while horizontal resorption increases the risk of cortical lingual plate perforation. All of these bone features make the lingual vessel closer and more easily damaged.

The majority of the literature describes [19] the mouth of floor hemorrhage as a consequence of dental implant placement with lingual cortical perforation and lingual vessel injury.

This complication happened in the first patient reported. The implants were inserted in knife edge mandibular ridge without any additional surgical procedure (bone grafting, GBR, and ridge augmentation), leading to lingual plate perforation, lingual vessel injury, and implant dehiscences and fenestrations. Probably, this lesion was not detected intraoperatively because of insufficient lingual flap harvesting.

Doubtless, it is a clear case of malpractice at various levels with ethical and legal implications. Faulty diagnosis, inadequate treatment plan, and probable surgical errors are involved [20].

In the second case, a GBR technique was correctly performed in order to place the fixture at the right vertical level to avoid increased crown length and to maintain adequate bone tissue (at least 1 mm) around the implants for a predictable outcome. The CT showed correct implant insertion within the bone without any lingual cortical plate perforation. The presence of abundant hematoma, mostly in the buccal area, suggested a submental ascending artery lesion as a cause of bleeding.

All bone reconstructive surgical techniques require a complete periosteal incision to increase the length of lingual and vestibular flaps in order to achieve a tension-free suture. This manoeuver can potentially damage the vessels on both lingual and buccal aspects because usually they run very close to the periosteum. This is what probably happened to this woman.

In both patients, the bleeding started some hours after surgery; as stated before, the local anesthesia may have hidden the vessel injury that provoked hemorrhage a few hours later. Therefore, the authors emphasize the importance of an observation period after surgery, which should last at least 2 h. Additionally, before discharge, the patient should be informed about this complication and advised to contact their doctor if it develops.

Generally, to prevent this complication or reduce the chance of occurrence, a preoperative CT is imperative, allowing for planning of the size and the angulation of each intended osseointegrated implant and revealing the position and diameter of intrabony vascular canals that may contain significant vessels, especially in the anterior mandible.

In the atrophic alveolar ridge, particular attention should be paid during implant surgery, adjusting the drilling angulation (buccolingually) to avoid lingual cortical plate perforation. An adequate surgical field with reflection of wide lingual and vestibular flaps helps to protect soft tissues and to identify any cortical perforation or bleeding sources. Accurate hemostasis after the periosteal incision is imperative to avoid this complication.

In addition, despite long implants being generally thought to be more reliable than short implants (more surface area contact and lower crown-to-implant ratios), several recent studies pointed out the diameter as a more important parameter compared to the length [21].

There is no need to use implants longer than 10 mm or even bicortical if inserted in a proper way. The use of short implants is strongly recommended in the anterior atrophic mandibular region in order to prevent bleeding complications [15, 22].

## 4. Conclusions

The presented report describes two cases of hemorrhage in the atrophic anterior mandible. In these conditions, implant surgery is challenging and requires more skills and attention because of the vessels' proximity and the limited amount of available bone.

In the first patient, the mouth floor hematoma was the consequence of lingual cortical plate perforation and injury of the lingual vessel, as widely described in the literature.

On the contrary, in the second patient, this potentially life-threatening complication was referred to as an injury of the buccal soft tissue artery during the periosteal release incision in the GBR technique.

Accordingly, in cases of bone reconstructive surgery (GBR in the case reported), which often requires periosteal release incisions, we emphasize meticulous hemostasis with electrocautery of both lingual and buccal flaps.

The diagnostic CT, required in all cases of implant surgery, is mandatory in demanding anatomic conditions such as anterior atrophic mandible, in which the risk of lingual plate perforation is increased and the vessel closer.

Wide subperiosteal undermining to better visualize mandibular bone plates, adjusting the drilling direction buccolingually, and the use of short implants are crucial to prevent this complication.

An observation period of 2 h after surgery is recommended for prompt detection of possible delayed bleeding. During this time, it is mandatory to be alert for hematomas on the floor of the mouth when patients complain of a protruding tongue, hemorrhage, or respiratory distress. Although no reports of death secondary to sublingual hematoma formation have been published, it is a potentially life-threatening complication, and airway security is the priority in these patients [21].

Further protocol and guidelines from experts are needed for surgery in the anterior mandible during implant placement.

## 5. Take Home Message

The mouth floor hemorrhage can happen not only for a cortical plate perforation during bone drilling and injury of a lingual vessel as reported by the literature. As sowed in this report, even the manipulation of buccal and lingual flaps mandatory in the GBR technique can lead to this complication. Prevention of adopting meticulous surgical techniques should be emphasized.

## Data Availability

The data used to support the findings of this study are included within the article.
